# Factors associated with change in moderate or severe symptoms of anxiety and depression in community-living adults and older adults during the COVID-19 pandemic

**DOI:** 10.17269/s41997-023-00832-y

**Published:** 2023-12-20

**Authors:** Helen-Maria Vasiliadis, Jessica Spagnolo, Mary Bartram, Marie-Josée Fleury, Jean-Philippe Gouin, Sébastien Grenier, Pasquale Roberge, Grace Shen-Tu, Jennifer E. Vena, Catherine Lamoureux-Lamarche, JianLi Wang

**Affiliations:** 1 Département des sciences de la santé communautaire, Université de Sherbrooke, Sherbrooke, Québec, Canada; 2 Centre de recherche Charles-Le Moyne, Longueuil, Québec, Canada; 3 Mental Health Commission of Canada, Ottawa, Ontario Canada; 4 School of Public Policy & Administration, Carleton University, Ottawa, Ontario Canada; 5 Douglas Mental Health University Institute, Verdun, Québec, Canada; 6 McGill University, Montreal, Québec, Canada; 7 Department of Psychology, Concordia University, Montreal, Québec, Canada; 8 Centre de recherche de l’Institut universitaire de gériatrie de Montréal, Montreal, Québec, Canada; 9 Département de psychologie, Université de Montréal, Montréal, Québec, Canada; 10 Département de médecine familiale et d’urgence, Université de Sherbrooke, Sherbrooke, Québec, Canada; 11 Centre de recherche du Centre hospitalier universitaire de Sherbrooke, Sherbrooke, Québec, Canada; 12 Alberta’s Tomorrow Project, Cancer Research & Analytics, Cancer Care Alberta, Alberta Health Services, Calgary, Alberta Canada; 13 Department of Community Health and Epidemiology, Dalhousie University, Halifax, Nova Scotia Canada

**Keywords:** Depression, Anxiety, Change in symptoms, Temporal patterns, Socio-economic, health and lifestyle behaviours, Psychosocial, COVID-19, Pandemic, Dépression, anxiété, changement de symptômes, patron temporel, facteurs socio-économiques, de santé et liés au mode de vie, psychosocial, COVID-19, pandémie

## Abstract

**Objectives:**

Few are the longitudinal studies on the changes in moderate or severe symptoms of anxiety or depression (MSS-ANXDEP) from before to during the COVID-19 pandemic in Canada. The aim was to study the change in MSS-ANXDEP and associated sociodemographic, economic, psychosocial, health behaviour and lifestyle, and clinical factors.

**Methods:**

The current sample includes 59,997 adults aged ≥ 35 years participating in the 2018 and 2020 health surveys of the 5 established cohorts of the Canadian Partnership for Tomorrow’s Health (CanPath). MSS-ANXDEP was based on a cutoff score ≥ 10 on the 7-item Generalized Anxiety Disorder Scale and Patient Health Questionnaire (PHQ-8). Change in MSS-ANXDEP was categorized as follows: no MSS-ANXDEP, remitted, incident, and persistent. Multinomial regressions were used to study MSS-ANXDEP as a function of sociodemographic, economic, psychosocial, health behaviours and lifestyle, and clinical factors.

**Results:**

Sociodemographic and economic (i.e. age, gender, cohort, race/ethnicity, lower income, decreased in income, work status, being an essential worker), lifestyle and health behaviours (i.e. smoking, cannabis and alcohol use, drinking more alcohol), psychosocial (i.e. provide help to others, information and instrumental support, and change in relationships with friends, family, and partner) and clinical factors (i.e. lifetime mental disorder and multimorbidity) were associated with remitted, incident, and persistent MSS-ANXDEP.

**Conclusion:**

Health and socio-economic factors were associated with changes in symptoms of anxiety and depression during the pandemic, further increasing inequities in mental health needs. Public health campaigns on the importance of healthy behaviours should continue and health policies should reduce economic and social barriers to integrated substance use and mental health care.

**Supplementary Information:**

The online version contains supplementary material available at 10.17269/s41997-023-00832-y.

## Résumé

**Objectifs:**

Les études longitudinales sur l’évolution des symptômes modérés ou sévères d’anxiété ou de dépression (SMS-ANXDEP) avant et pendant la pandémie de COVID-19 au Canada sont rares. L’objectif était d’étudier l’association entre l’évolution des SMS-ANXDEP et les facteurs sociodémographiques, économiques, psychosociaux, cliniques et liés aux comportements et au mode de vie, avant et pendant la pandémie.

**Méthodes:**

Ce grand échantillon comprend 59 997 adultes âgés de ≥ 35 ans qui ont participé aux enquêtes de santé 2018 et 2020 des 5 cohortes établies du Partenariat canadien pour la santé de demain (CanPath). La présence de SMS-ANXDEP a été définie par un résultat ≥ 10 sur les échelles *Generalized Anxiety Disorder Scale* à 7 items (GAD-7) et *Patient Health Questionnaire* (PHQ-8). Les changements dans les SMS-ANXDEP ont été catégorisés selon les patrons temporels suivants : absence, rémission, incidence et persistance de SMS-ANXDEP. Des régressions multinomiales multivariées ont été utilisées pour étudier les patrons temporels de SMS-ANXDEP en fonction, des facteurs socio-démographiques, économiques, associés au style de vie et aux comportements de santé, psychosociaux et cliniques.

**Résultats:**

Les facteurs socio-démographiques et économiques (âge, genre, cohorte, race/ethnie, revenu inférieur, diminution du revenu, statut d’emploi, être un travailleur essentiel), associés au style de vie et aux comportements de santé (tabagisme, consommation de cannabis et d’alcool, consommation accrue d’alcool), psychosociaux (offrir de l’aide pendant la pandémie, soutien en information et instrumental, changement dans les relations avec les amis, la famille et la personne partenaire) et cliniques (trouble mental au cours de la vie, multimorbidité) étaient associés à la présence de SMS-ANXDEP en rémission, incident et persistant.

**Conclusion:**

Les patrons temporels des SMS-ANXDEP pendant la pandémie étaient associés aux facteurs socio-économiques et de santé, suggérant des inégalités accrues en matière de besoins de santé mentale. Les campagnes de santé publique sur l’importance d’adopter des comportements sains devraient continuer et les politiques de santé devraient réduire les barrières économiques et sociales aux soins intégrés de santé mentale et de toxicomanie.

## Introduction

Mental health needs increased in the context of the COVID-19 pandemic (World Health Organization, 2022). SARS-CoV-2 infection rates and decreased mobility due to public health physical distancing mandates were associated with major depressive and anxiety disorders (COVID-19 Mental Disorders Collaborators, 2021). Higher prevalence in self-reported anxiety and depression in general population studies during the pandemic as compared to pre-pandemic periods was observed across 49 countries, with meta-regression prevalence differences estimated at 27.6% and 25.6%, respectively (COVID-19 Mental Disorders Collaborators, 2021). Data from a recent Dutch population–based study showed an increase in overall prevalence of mental and substance use disorders between 2010–2012 and 2016–2018, which continued to increase in 2019–2022 (Ten Have et al., 2023). In Canada, in comparison to the month before the pandemic, a cross-sectional survey showed a 12% and 29% increase in reported mild to severe symptoms of generalized anxiety and depression in individuals without a history of mental illness (Robillard et al., 2021). In one of the rare longitudinal studies on pre-post COVID changes amid the same population sampled, the Canadian Longitudinal Study on Aging (CLSA) showed an increase in moderate to severe symptoms of depression from 16.4% during the pre-COVID-19 phase to 22.0% during the first pandemic wave in older adults (Raina et al., 2021).

Studies also reported on the factors associated with anxiety and depression during the COVID-19 pandemic. Gender, racial, and socio-economic inequities related to mental health status were observed (Chen & Wang, 2021; Public Health Agency of Canada, 2021). Descriptive statistics in Canada showed that identifying as a woman, Indigenous, or a healthcare worker, as well as reporting decreased income due to the pandemic, lifetime traumatic events, or chronic conditions, were associated with a higher prevalence of anxiety and depression at the start of the pandemic (Public Health Agency of Canada, 2020; Statistics Canada, 2021). Individuals infected with the SARS-CoV-2 virus (Inchausti et al., 2020) were also more likely to report anxiety and depression.

Social and economic disruptions related to the COVID-19 pandemic posed a risk to marital satisfaction (Carrese-Chacra et al., 2023) and the well-being of families and communities (Prime et al., 2020). A recent study on Dutch older adults aged 55 years and over showed that regardless of pre-pandemic mental health, size of network was associated with symptoms of anxiety during the pandemic (Holwerda et al., 2023). A study during the COVID-19 pandemic focusing on the change in quality of relationships between married adults with children also showed a steeper decline in satisfaction with the relationship in women and in individuals with symptoms of depression (James et al., 2022).

Depression and anxiety disorders are associated with increased functional limitations and decreased quality of life and loss in productivity (Chisholm et al., 2016; COVID-19 Mental Disorders Collaborators, 2021; Hohls et al., 2021). Given that mental health unmet needs increased in the past decade (Ten Have et al., 2023) and in the context of the COVID-19 pandemic (World Health Organization, 2022), it is important to identify individuals at risk for new and persistent symptoms of anxiety and depression as well as the factors associated with the course of depression and anxiety symptoms (i.e., persistence, incidence, remission) to inform on better treatment plans. Scarce are the longitudinal studies that have assessed the factors associated with the course of depression and anxiety in general populations while accounting for income prior to the pandemic and changes in socio-economic, employment, and psychosocial factors and health behaviours during the pandemic, in Canada and elsewhere. Further, although the importance to report sex- and age-specific results has been highlighted (Institute of Medicine (US) Board on Population Health and Public Health Practice, 2012; Dannefer, 2001; Abramson & Portacolone, 2017), studies have not reported age- and sex-specific factors associated with temporal patterns of depression and anxiety disorders, which can inform on the social and structural inequities leading to anxiety and depression.

The current study objectives aimed to fill this gap by assessing the temporal patterns (absence, remission, incidence, persistence) of moderate to severe symptoms of anxiety and depression in adults and older adults from before to during the first two waves of the COVID-19 pandemic across Canada. The COVID-19 pandemic had an impact on the risk of infection and the health of populations, on psychological and behavioural factors, as well as on socio-economic factors. The biopsychosocial model was therefore used as a guiding framework to study the distinct contributions of social determinants of health, psychological factors, and medical and health-related behaviours on the temporal patterns of anxiety and depression. Also identified in a conceptual framework for public mental health (Dykxhoorn et al., 2022), the roles of a range of socio-economic, psychological, and health and lifestyle behaviour correlates associated with the temporal patterns of anxiety and depression will be studied. This can be useful in informing population and public health (Bolton & Gillett, 2019; Frazier, 2020). Therefore, as a second aim, the roles of a range of sociodemographic, economic, employment, psychosocial, health behaviour, and lifestyle factors, as well as clinical factors as correlates, will be studied. Although the pandemic has evolved, recent data show that improvements in mental health have stagnated and are still at much higher levels than pre-pandemic (Mental Health Research Canada, 2023). As the effect of the pandemic on mental health is proving to be persistent, the current study focusing on the first two waves of the pandemic captures an important period where we can identify at-risk individuals whose mental health worsened and assess the factors that may further disadvantage these individuals following pandemic peaks. This period was associated with intense public health and physical distancing restrictions without effective vaccines, potentially contributing to changes in mental health. The equitable distribution of health is a priority for population health and value-based health systems (Teisberg et al., 2020; Smith et al., 2021). This study will aim to characterize at-risk individuals with persistent and onset of new symptoms of anxiety and depression. The findings can inform public health programs to ameliorate mental health in better tailoring healthier lifestyle promotion campaigns. Further, the findings can inform health policies aimed at improving the efficient allocation of resources for equitable access to integrated substance use and mental health care to adequately address population mental health needs.

## Methods

### Study population and procedures

The current study draws on harmonized survey data made available by CanPath—the Canadian Partnership for Tomorrow’s Health (formerly Canadian Partnership for Tomorrow Project, CPTP), and its 5 established regional cohorts, namely the British Columbia Generations Project (BCGP), Alberta’s Tomorrow Project (ATP), Ontario Health Study (OHS), Quebec’s CARTaGENE (CaG), and Atlantic Partnership for Tomorrow’s Health (Atlantic PATH). The recruitment and profile of CanPath participants have been described elsewhere (Dummer et al., 2018). In general, CanPath participants were more likely to be female, report higher income and education, be retired, and self-report as White than the general Canadian population (Dummer et al., 2018). The current study relied on data from 69,143 participants who had responded to both the 2018 CanPath and the COVID-19 follow-up questionnaire (May–December 2020) (https://canpath.ca/en/covid-19-initiatives). The temporal patterns of moderate or severe symptoms of anxiety or depression were assessed at both surveys, while all the other independent study variables were assessed during the pandemic 2020 survey, except for race/ethnicity, which was ascertained in the survey prior to the pandemic. The present study sample includes *n* = 59,997 (86.8%) participants (22.6% from BCGP, 12.5% from ATP, 40.6% from OHS, 8.9% from CaG, 15.4% from PATH) with complete data. The study was approved by the institutional research ethics board of the CISSS Montérégie-Centre (#2021-563).

### Measures

#### Dependent variables of interest

##### Temporal patterns of moderate or severe symptoms of either anxiety or depression (MSS-ANXDEP) during the pandemic

Anxiety was assessed at both surveys with the 7-item Generalized Anxiety Disorder Scale (GAD-7) (score range 0–21) (Spitzer et al., 2006). A cutoff score of ≥ 10 has been recommended as a positive screen for anxiety representing moderate and severe symptoms. Depression was based on a cutoff score of ≥ 10 on 8 items of the Patient Health Questionnaire (PHQ-8) (score range 0–24), representing moderate to severe depressive symptoms (Kroenke et al., 2009). Information from both 2018 and 2020 (May–Dec 2020) surveys was considered to create a four-level mutually exclusive dummy variable that would summarize the presence of moderate or severe symptoms of either anxiety or depression (MSS-ANXDEP) at each survey: no MSS at either survey; remitted (MSS present in 2018, pre-COVID, but not present in 2020); incident (MSS not present in 2018 but was present in 2020); or persistent (MSS present in both 2018 and 2020).

#### Independent variables

##### Sociodemographic and economic factors

Sociodemographic factors assessed included age categorized into four groups (35–44 years, 45–54 years, 55–64 years, ≥ 65 years), gender (male, female, other gender diverse groups), and race/ethnicity (yes, self-identified as White, or no, self-identified as Arab, Black, East Asian, Filipino, Jewish, Latin American/Hispanic, South Asian, South-East Asian, West-Asian, Other). The CanPath regional cohort that individuals belonged to was also considered. Economic factors assessed total household income before taxes in the previous year (categorized as < $24,999, $25,000–$49,999, $50,000–$74,999, $75,000–$99,999, $100,000–$149,999, ≥ $150,000; or prefer not to respond, doesn’t know/missing); decrease in income during the pandemic (yes/no); loss of job during the pandemic (yes/no); and work status (categorized as full-time or part-time employed/self-employed, retired, looking after home and/or family/doing unpaid or voluntary work/student, unable to work because of sickness or disability, unemployed, prefer not to answer/missing data). Respondents were also asked whether they worked as a medical professional, as well as category of professional with exposure to patients (e.g., physician, nurse, hospital employee, first responder, pharmacist) (yes/no) and whether they worked as an essential service provider with regular exposure to members of the public (e.g., grocery store attendant, public transit, police, security) (yes/no).

##### Lifestyle and health behaviour factors

Lifestyle habits included past-month smoking (occasionally/daily or not at all), the presence of past-year cannabis use (yes or no), and past-year average weekly alcohol consumption (never, less than daily (≤ 5 times a week), or almost daily (6–7 times a week)).

##### Psychosocial factors

Factors included providing since March 2020 any kind of help to others (e.g., emotional/psychological, medical, informational, financial, practical, other) because of the pandemic (yes/no); receipt of informational, financial, or practical support (e.g., housing, childcare, food delivery, material goods furniture, clothing; yes/no); and change in relationship with friends, family, and partner (categorized as follows: more distant or strained than before the pandemic, about the same as before the pandemic, has become closer than before the pandemic, prefer not to respond/missing data).

##### Clinical factors

Self-reported lifetime diagnosis of a mental disorder was based on whether a doctor had ever told the respondent that they had a mental health condition and categorized as yes, no, or not answered/not reported. Physical multimorbidity was based on self-reported lifetime physician diagnosis of physical disorders (e.g., cancer, diabetes, heart and circulatory conditions, cardiovascular disorder, respiratory system conditions, gastrointestinal diseases, liver or pancreatic conditions, renal disease, kidney conditions, neurological conditions, bone and joint conditions, and immune system conditions) and categorized as no (0) chronic physical or neurologic condition, 1 to 2 chronic conditions, or ≥ 3 chronic conditions.

### Data analyses

Descriptive statistics and group comparisons were based on chi-square statistics. Multivariable multinomial regression analyses were carried out to study the outcome change of anxiety and depression as a function of sociodemographic and economic, lifestyle and health behaviour, psychosocial, and clinical factors. Analyses were stratified according to gender (female, male) and age group (< 65 years, ≥ 65 years). Multicollinearity between variables was not observed. Adjusted odds ratios (AOR) and 95% confidence intervals (CI) were computed to determine the strength of associations. Differences observed in stratified analyses were considered significant if 95% confidence intervals did not overlap. Analyses were carried out using SAS version 9.4 (SAS Institute 2013).

## Results

Most of the sample identified as White (93.6%) and female (65.0%), and were aged 65 years and older (47.9%) (Table 1). Characteristics of the study sample according to the temporal patterns in MSS-ANXDEP are presented in Table 1. The proportion of participants reporting the absence of MSS-ANXDEP at either survey was 85.7% and of those reporting remitted, incident, and persistent MSS-ANXDEP was 5.2%, 5.7%, and 3.4%, respectively.

**Table 1:** Characteristics of study sample by temporal patterns of moderate or severe symptoms of either anxiety or depression

		Patterns of MSS of anxiety and depression (*n* = 59,997)				
	Overall *N* = 59,997	No disorder *N* = 51,446 (85.7%)	Remitted *N* = 3123 (5.2%)	Incident *N* = 3398 (5.7%)	Persistent *N* = 2030 (3.4%)	*p*-value
***Sociodemographic and economic factors***						
Age group						<0.0001
≤ 44 years	2591 (4.3%)	1856 (71.7%)	198 (7.6%)	308 (11.9%)	229 (8.8%)	
45–54 years	9259 (15.4%)	7198 (77.7%)	665 (7.2%)	812 (8.8%)	584 (6.3%)	
55–64 years	19,420 (32.4%)	16,370 (84.3%)	1135 (5.9%)	1208 (6.2%)	707 (3.6%)	
≥ 65 years	28,727 (47.9%)	26,022 (90.6%)	1125 (3.9%)	1070 (3.7%)	510 (1.8%)	
Gender identity						<0.0001
Male	20,832 (34.7%)	18,893 (90.7%)	864 (4.1%)	658 (3.2%)	417 (2.0%)	
Female	39,002 (65.0%)	32,433 (83.2%)	2244 (5.7%)	2724 (7.0%)	1601 (4.1%)	
Gender diverse groups	163 (0.3%)	120 (73.6%)	15 (9.2%)	16 (9.8%)	12 (7.4%)	
Self-reported as White						0.02
Yes	56,181 (93.6%)	48,198 (85.8%)	2884 (5.1%)	3194 (5.7%)	1905 (3.4%)	
No	3816 (6.4%)	3248 (85.1%)	239 (6.3%)	204 (5.3%)	125 (3.3%)	
Income prior to pandemic						<0.0001
Less than $24,999	1943 (3.2%)	1380 (71.0%)	207 (10.7%)	151 (7.8%)	205 (10.5%)	
$25,000–$49,999	6608 (11.0%)	5440 (82.3%)	444 (6.7%)	413 (6.3%)	311 (4.7%)	
$50,000–$74,999	9844 (16.4%)	8466 (86.0%)	514 (5.2%)	541 (5.5%)	323 (3.3%)	
$75,000–$99,999	9547 (15.9%)	8286 (86.8%)	444 (4.6%)	521 (5.5%)	296 (3.1%)	
$100,000–$149,999	11,579 (19.3%)	10,020 (86.5%)	534 (4.6%)	668 (5.8%)	357 (3.1%)	
$150,000 or more	10,671 (17.8%)	9288 (87.0%)	470 (4.4%)	638 (6.0%)	275 (2.6%)	
Prefer not to respond	9251 (15.4%)	8117 (87.7%)	468 (5.1%)	423 (4.6%)	243 (2.6%)	
Missing response	554 (1.0%)	449 (81.0%)	42 (7.6%)	43 (7.8%)	20 (3.6%)	
Decrease in income during pandemic						<0.0001
Yes	14,037 (23.4%)	11,381 (81.1%)	881 (6.3%)	1106 (7.9%)	669 (7.8%)	
No	45,960 (76.6%)	40,065 (87.2%)	2242 (4.9%)	2292 (5.0%)	1361 (2.9%)	
Loss of job during the pandemic						<0.0001
Yes	3270 (5.5%)	2642 (80.8%)	210 (6.4%)	266 (8.1%)	152 (4.7%)	
No	56,727 (94.5%)	48,804 (86.0%)	2913 (5.2%)	3132 (5.5%)	1878 (3.3%)	
Work status						<0.0001
Full-time or part-time employed/self-employed	24,838 (41.4%)	20,510 (82.6%)	1489 (6.0%)	1798 (7.2%)	1041 (4.2%)	
Retired	26,666 (44.4%)	24,018 (90.1%)	1095 (4.1%)	1035 (3.9%)	518 (1.9%)	
Looking after home and/or family/doing unpaid or voluntary work/student	1592 (2.7%)	1305 (82.0%)	103 (6.5%)	111 (7%)	73 (4.6%)	
Unable to work because of sickness/disability	959 (1.6%)	441 (46.0%)	130 (13.5%)	134 (14.0%)	254 (26.5%)	
Unemployed	515 (0.9%)	374 (72.6%)	57 (11.1%)	50 (9.7%)	34 (6.6%)	
Prefer not to answer/missing data	5427 (9.0%)	4798 (88.4%)	249 (4.6%)	270 (5.0%)	110 (2.0%)	
***Lifestyle and health behaviour factors***						
Current smoking						<0.0001
Yes	2542 (4.2%)	1893 (74.5%)	203 (8.0%)	224 (8.8%)	222 (8.7%)	
No	57,455 (95.8%)	49,553 (86.2%)	2920 (5.1%)	3174 (5.5%)	1808 (3.2%)	
Past-year cannabis use						<0.0001
Yes	8071 (13.5%)	6133 (76.0%)	560 (7.0%)	802 (9.9%)	576 (7.1%)	
No	51,926 (86.5%)	45,313 (87.3%)	2563 (4.9%)	2596 (5.0%)	1454 (2.8%)	
Past-year average weekly alcohol consumption						<0.0001
Never	6813 (11.4%)	5541 (81.3%)	454 (6.7%)	447 (6.6%)	371 (5.4%)	
Less than daily (≤ 5 times/week)	44,549 (74.2%)	38,315 (86.0%)	2300 (5.2%)	2502 (5.6%)	1432 (3.2%)	
Almost daily (6–7 times a week)	8635 (14.4%)	7590 (87.9%)	369 (4.3%)	449 (5.2%)	227 (2.6%)	
Drinking alcohol more often than prior to March 2020						<0.0001
Yes	10,197 (17.0%)	8147 (79.9%)	577 (5.6%)	976 (9.6%)	497 (4.9%)	
No	49,800 (83.0%)	43,299 (86.9%)	2546 (5.1%)	2422 (4.9%)	1533 (3.1%)	
Medical professional						<0.0001
Yes	3563 (5.9%)	2932 (82.3%)	201 (5.6%)	272 (7.6%)	158 (4.4%)	
No	56,434 (94.1%)	48,514 (86.0%)	2922 (5.2%)	3126 (5.5%)	1872 (3.3%)	
Essential worker						<0.0001
Yes	3585 (6.0%)	2863 (79.9%)	246 (6.9%)	299 (8.3%)	177 (4.9%)	
No	56,412 (94.0%)	48,583 (86.1%)	2877 (5.1%)	3099 (5.5%)	1853 (3.3%)	
***Psychosocial factors***						
Provide help to others because of the pandemic						<0.0001
Yes	38,477 (64.1%)	32,971 (85.7%)	1879 (4.9%)	2342 (6.1%)	1285 (3.3%)	
No	21,520 (35.9%)	18,475 (85.8%)	1244 (5.8%)	1056 (4.9%)	745 (3.5%)	
Receipt of informational/financial/practical support						<0.0001
Yes	15,196 (25.3%)	12,498 (82.3%)	779 (5.1%)	1237 (8.1%)	682 (4.5%)	
No	44,801 (74.7%)	38,948 (87.0%)	2344 (5.2%)	2161 (4.8%)	1348 (3.0%)	
Change in relationship with friends						<0.0001
More distant or strained than before the pandemic	12,724 (21.2%)	9819 (77.2%)	760 (6.0%)	1291 (10.1%)	854 (6.7%)	
About the same as before the pandemic	40,548 (67.6%)	36,066 (89.0%)	1954 (4.8%)	1635 (4.0%)	893 (2.2%)	
Has become closer than before the pandemic	5372 (8.9%)	4506 (83.9%)	291 (5.4%)	381 (7.1%)	194 (3.6%)	
Not applicable/not reported	1353 (2.3%)	1055 (78.0%)	118 (8.7%)	91 (6.7%)	89 (6.6%)	
Change in relationship with family						<0.0001
More distant or strained than before the pandemic	5510 (9.2%)	3953 (71.7%)	370 (6.7%)	697 (12.7%)	490 (8.9%)	
About the same as before the pandemic	40,798 (68.0%)	35,970 (88.2%)	1988 (4.9%)	1770 (4.3%)	1070 (2.6%)	
Has become closer than before the pandemic	10,750 (17.9%)	9058 (84.3%)	564 (5.2%)	765 (7.1%)	363 (3.4%)	
Not applicable/not reported	2939 (4.9%)	2465 (83.9%)	201 (6.8%)	166 (5.7%)	107 (3.6%)	
Change in relationship with partner						<0.0001
More distant or strained than before the pandemic	3857 (6.4%)	2534 (65.7%)	273 (7.1%)	647 (16.8%)	403 (10.4%)	
About the same as before the pandemic	34,173 (57.0%)	30,616 (89.6%)	1552 (4.5%)	1305 (3.8%)	700 (2.1%)	
Has become closer than before the pandemic	9446 (15.7%)	8083 (85.6%)	486 (5.1%)	591 (6.3%)	286 (3.0%)	
Not applicable/not reported	12,521 (20.9%)	10,213 (81.6%)	812 (6.5%)	855 (6.8%)	641 (5.1%)	
***Clinical factors***						
Lifetime physician diagnosis of a mental disorder						<0.0001
No	41,574 (69.3%)	38,330 (92.2%)	1399 (3.4%)	1423 (3.4%)	422 (1.0%)	
Yes	13,018 (21.7%)	8341 (64.1%)	1471 (11.3%)	1707 (13.1%)	1499 (11.5%)	
Not answered	5405 (9.0%)	4775 (88.3%)	253 (4.7%)	268 (5.0%)	109 (2.0%)	
The presence of multimorbidity						<0.0001
No chronic physical conditions	17,072 (28.5%)	15,218 (89.1%)	713 (4.2%)	806 (4.7%)	335 (2.0%)	
1–2 chronic physical conditions	39,124 (65.2%)	33,484 (85.6%)	2064 (5.3%)	2242 (5.7%)	1334 (3.4%)	
≥3 chronic physical conditions	3801 (6.3%)	2744 (72.2%)	346 (9.1%)	350 (9.2%)	361 (9.5%)	

Results from the multivariable analyses in the overall sample are presented in Table 2. Overall, as compared to older adults aged ≥ 65 years, individuals aged 35–44 years, those aged 45–54 years, and those aged 55–64 years were more likely to experience remitted, incident, and persistent MSS-ANXDEP. As compared to males, females and other gender diverse groups also experienced a change in symptoms and had remitted, incident, and persistent MSS-ANXDEP. Individuals self-declaring as White were less likely to experience remission in MSS-ANXDEP.

**Table 2:** Multivariable analyses of temporal patterns of moderate or severe symptoms of either anxiety or depression and study factors

	Depression or anxiety Moderate or severe symptoms								
	Remitted versus no disorder			Incident versus no disorder			Persistent versus no disorder		
	AOR	95% CI		AOR	95% CI		AOR	95% CI	
***Sociodemographic and economic factors***									
Age groups									
35–44 years	**2.194**	**1.815**	**2.652**	**2.383**	**2.003**	**2.835**	**4.323**	**3.460**	**5.401**
45–54 years	**1.970**	**1.727**	**2.249**	**1.888**	**1.657**	**2.151**	**3.172**	**2.665**	**3.774**
55–64 years	**1.419**	**1.281**	**1.572**	**1.389**	**1.251**	**1.541**	**1.621**	**1.401**	**1.875**
≥ 65 years	**Reference**			**Reference**			**Reference**		
Gender identity									
Male	**Reference**			**Reference**			**Reference**		
Female	**1.256**	**1.152**	**1.370**	**1.986**	**1.807**	**2.183**	**1.585**	**1.402**	**1.792**
Gender diverse groups	**1.891**	**1.079**	**3.315**	**2.706**	**1.533**	**4.776**	**2.427**	**1.226**	**4.807**
Self-reporting as White (yes vs no)	**0.797**	**0.690**	**0.921**	0.998	0.853	1.167	0.904	0.737	1.109
Income prior to pandemic									
Less than $24,999	**2.487**	**2.040**	**3.032**	**1.244**	**1.004**	**1.541**	**2.808**	**2.203**	**3.579**
$25,000–$49,999	**1.822**	**1.564**	**2.122**	**1.219**	**1.049**	**1.418**	**2.044**	**1.674**	**2.497**
$50,000–$74,999	**1.412**	**1.227**	**1.626**	1.099	0.960	1.257	**1.471**	**1.218**	**1.776**
$75,000–$99,999	**1.254**	**1.090**	**1.444**	1.108	0.971	1.263	**1.474**	**1.225**	**1.772**
$100,000–$149,999	**1.162**	**1.019**	**1.325**	1.082	0.960	1.221	**1.341**	**1.129**	**1.594**
$150,000 or more	**Reference**			**Reference**			**Reference**		
Prefer not to respond	**1.435**	**1.245**	**1.654**	1.053	0.915	1.212	**1.527**	**1.254**	**1.859**
Missing response	**1.988**	**1.411**	**2.801**	**1.467**	**1.038**	**2.073**	**1.725**	**1.043**	**2.851**
Decrease in income during pandemic	**1.193**	**1.087**	**1.308**	**1.271**	**1.163**	**1.388**	**1.301**	**1.159**	**1.461**
Loss of job during the pandemic	0.959	0.813	1.131	0.877	0.751	1.023	0.867	0.708	1.062
Work status									
Full-time or part-time employed/self-employed	**Reference**			**Reference**			**Reference**		
Retired	**0.859**	**0.767**	**0.961**	0.896	0.800	1.004	0.873	0.745	1.022
Looking after home and/or family/doing unpaid or voluntary work/student	1.029	0.827	1.281	0.948	0.763	1.178	1.026	0.781	1.348
Unable to work because of sickness/disability	**1.872**	**1.499**	**2.337**	**1.840**	**1.472**	**2.301**	**3.562**	**2.882**	**4.401**
Unemployed	**1.524**	**1.127**	**2.060**	1.292	0.935	1.785	1.198	0.806	1.781
Prefer not to answer/missing data	1.694	0.913	3.144	1.268	0.626	2.567	1.341	0.572	3.143
Medical professional	0.908	0.776	1.062	0.983	0.853	1.133	1.018	0.844	1.228
Essential worker	**1.178**	**1.019**	**1.361**	**1.373**	**1.196**	**1.576**	**1.229**	**1.026**	**1.473**
***Lifestyle and health behaviours factors***									
Current smoking	**1.262**	**1.077**	**1.478**	**1.244**	**1.064**	**1.454**	**1.660**	**1.396**	**1.974**
Past-year cannabis use	**1.204**	**1.088**	**1.333**	**1.564**	**1.426**	**1.716**	**1.590**	**1.415**	**1.787**
Past-year average weekly alcohol consumption									
Never	**Reference**			**Reference**			**Reference**		
Less than daily (≤ 5 times/week)	**0.870**	**0.778**	**0.972**	**0.764**	**0.681**	**0.857**	**0.669**	**0.583**	**0.769**
Almost daily (6–7 times a week)	0.866	0.743	1.009	**0.843**	**0.725**	**0.981**	**0.759**	**0.623**	**0.924**
Drinking alcohol more often than prior to March 2020	**1.112**	**1.005**	**1.229**	**1.617**	**1.481**	**1.766**	**1.463**	**1.296**	**1.652**
***Psychosocial factors***									
Provide help to others because of the pandemic	**0.835**	**0.772**	**0.903**	0.991	0.913	1.075	**0.852**	**0.768**	**0.946**
Receipt of informational/financial/practical support	**0.830**	**0.759**	**0.908**	**1.333**	**1.230**	**1.446**	1.023	0.917	1.141
Change in relationship with friends									
More distant or strained than before the pandemic	**Reference**			**Reference**			**Reference**		
About the same as before the pandemic	**0.860**	**0.784**	**0.945**	**0.501**	**0.460**	**0.545**	**0.448**	**0.401**	**0.501**
Has become closer than before the pandemic	0.918	0.790	1.067	**0.639**	**0.560**	**0.730**	**0.602**	**0.502**	**0.721**
Not applicable/not reported	1.240	0.985	1.560	**0.717**	**0.559**	**0.919**	0.839	0.634	1.109
Change in relationship with family									
More distant or strained than before the pandemic	**Reference**			**Reference**			**Reference**		
About the same as before the pandemic	**0.764**	**0.674**	**0.866**	**0.509**	**0.458**	**0.566**	**0.483**	**0.423**	**0.551**
Has become closer than before the pandemic	**0.729**	**0.628**	**0.845**	**0.596**	**0.527**	**0.675**	**0.439**	**0.372**	**0.518**
Not applicable/not reported	0.889	0.728	1.087	**0.589**	**0.483**	**0.719**	**0.441**	**0.341**	**0.568**
Change in relationship with partner									
More distant or strained than before the pandemic	**Reference**			**Reference**			**Reference**		
About the same as before the pandemic	**0.633**	**0.550**	**0.729**	**0.270**	**0.241**	**0.302**	**0.259**	**0.223**	**0.299**
Has become closer than before the pandemic	**0.668**	**0.569**	**0.785**	**0.340**	**0.299**	**0.387**	**0.302**	**0.253**	**0.361**
Not applicable/not reported	**0.699**	**0.599**	**0.815**	**0.392**	**0.345**	**0.445**	**0.438**	**0.374**	**0.514**
***Clinical factors***									
Lifetime physician diagnosis of a mental disorder									
No	**Reference**			**Reference**			**Reference**		
Yes	**3.910**	**3.607**	**4.238**	**3.807**	**3.517**	**4.120**	**9.834**	**8.752**	**11.050**
Not answered	**6.881**	**3.906**	**12.121**	**2.732**	**1.253**	**5.959**	**6.544**	**2.688**	**15.930**
The presence of multimorbidity									
No chronic physical conditions	**Reference**			**Reference**			**Reference**		
1–2 chronic physical conditions	**1.336**	**1.218**	**1.464**	**1.342**	**1.226**	**1.468**	**1.779**	**1.558**	**2.030**
≥ 3 chronic physical conditions	**2.287**	**1.974**	**2.649**	**2.149**	**1.853**	**2.492**	**4.133**	**3.443**	**4.962**

Bold estimates: *p* < 0.05

*AOR*, adjusted odds ratios. Models adjusted for study factors and regional cohorts

As compared to individuals reporting pre-pandemic income in the ≥ $150,000 bracket, those reporting income in all other lower income brackets were more likely to experience remitted and persistent MSS-ANXDEP, whereas those reporting pre-pandemic income in the < $25,000 and $25,000–$49,999 brackets were more likely to experience incident MSS-ANXDEP. A decrease in income during the pandemic was associated with change in MSS-ANXDEP, whereas loss of employment during the pandemic was not.

Individuals who were unable to work due to illness or disability were more likely to experience remitted, incident, and persistent MSS-ANXDEP. Individuals who reported being unemployed were also more likely to experience remitted MSS-ANXDEP. Individuals looking after home and/or family, doing unpaid or voluntary work, or being a student did not experience changes in MSS-ANXDEP. Essential workers who reported contact with the public were more likely to experience remitted, incident, and persistent MSS-ANXDEP. Medical professionals did not experience a change in MSS-ANXDEP.

Individuals reporting current smoking, past-year cannabis use, and drinking alcohol more often since the pandemic were more likely to experience remitted, incident, and persistent MSS-ANXDEP.

Individuals providing help to others because of the pandemic were less likely to experience persistent MSS-ANXDEP. Individuals receiving informational, financial, or practical support during the pandemic were more likely to report incident MSS-ANXDEP. Individuals reporting a more distant or strained relationship than before the pandemic with friends, family, and partner experienced remitted, incident, and persistent MSS-ANXDEP.

Individuals reporting a lifetime physician diagnosis of a mental disorder and multimorbidity were more likely to experience remitted, incident, and persistent MSS-ANXDEP.

Results from the multivariable analyses restricted to participants aged < 65 years and ≥ 65 years are presented in Supplementary Tables 1 and 2. In general, the temporal patterns of MSS-ANXDEP observed in the overall sample are similar in individuals aged < 65 years and those ≥ 65 years with few exceptions. Older adults aged ≥ 65 years also reporting a lifetime physician diagnosis of a mental disorder were more likely to experience incident MSS-ANXDEP and multimorbidity than adults aged < 65 years. Results from the multivariable analyses restricted to females and males are presented in Supplementary Tables 3 and 4. In general, the factors associated with the temporal patterns of MSS-ANXDEP observed in the overall sample are similar in females and males. Of interest, females who reported current smoking were more likely to experience MSS-ANXDEP.

## Discussion

This study contributes to the present literature by documenting the temporal patterns of MSS of either anxiety or depression in a large sample of adults and older adults across Canada from before to during the first two waves of the pandemic and reporting on associated factors in the overall sample and restricted by age groups and gender. The prevalence of absent, remitted, incident, and persistent MSS of either anxiety or depression in the current study was 85.7%, 5.2%, 5.7%, and 3.4%, respectively. A Norwegian cohort study similarly showed pre-pandemic prevalence of mental disorders of 15.4% and ranging from 9.0% to 14.3% during the early phases of the pandemic (March to September 2020) (Knudsen et al., 2022). Our study findings are also similar to a longitudinal German cohort study which highlights that among those who screened positive for depression in 2019, 33.6% and 21.9% also screened positive for depression and anxiety in 2021, whereas among those who did not screen positive for depression in 2019, 9.8% and 6.5% screened positive for depression and anxiety in 2021 (Benke et al., 2022). Longitudinal studies with increased number of follow-ups including measures before and during the pandemic are needed to better describe the temporal patterns of anxiety and depression.

Priorities for value-based health systems include equitable distribution of health regardless of ethnicity, income, or place of residence (Smith et al., 2021). Older adults in the current study were less likely to experience MSS-ANXDEP, which has been similarly reported elsewhere (Public Health Agency of Canada, 2022). However, the stratified analyses showed that older adults with a lifetime physician diagnosis of a mental disorder were more likely to experience MSS-ANXDEP during the pandemic than younger adults aged < 65 years. Factors such as increased isolation during the pandemic in older adults may in part explain this finding (Lara et al., 2023). The current study highlighted potential socio-economic inequities in the temporal patterns of anxiety and depression, even after considering health status and sociodemographic factors and health and lifestyle behaviours. Findings showed that individuals experiencing persistent MSS-ANXDEP had lower income levels and reported a decrease in income during the pandemic. These findings may hint at the links between financial insecurity and mental health status (Hertz-Palmor et al., 2021) and the effect on perpetuating pre-existing social and economic inequities (Asmundson et al., 2020). Findings related to income (i.e., income prior to the pandemic and reporting a decrease in income since the pandemic) and its influence on incident and/or persistent MSS-ANXDEP are important to consider in the context of public health practice and policy. They can encourage practitioners to ask about changes in socio-economic factors during consultation, as well as inform decision- and policy-makers on allocation to mental health services and social security nets.

Findings from the current study also showed that essential workers experienced remitted, incident, and persistent MSS-ANXDEP. These findings suggest that the population of essential workers, which included professions working in direct contact with the public, may have been at greater risk of new anxiety and depression symptoms during the pandemic context, which encourages the need for access to mental health interventions for this specific population of workers.

Individuals experiencing MSS-ANXDEP also reported current smoking, cannabis use in the past year, and an increase in alcohol consumption since the pandemic, and this was similarly observed in adults and older adults, and females and males. To our knowledge, rare are the studies that have assessed age and gender differences in anxiety or depression in the context of the COVID-19 pandemic. Similar to our findings, one of the rare studies identified showed that smoking was associated with recurrent depression in females (Tibubos et al., 2019). A recent Canadian study showed a relationship between burnout and dependency to alcohol and/or cannabis suggesting their use as potential coping mechanisms (Mental Health Research Canada, 2023). Public health campaigns should reiterate the negative health effects of behaviours such as smoking, cannabis use, and alcohol consumption on health and mental health and provide access to integrated mental health support programs aimed at addressing multifaceted mental health needs.

In the current study, individuals experiencing incident MSS-ANXDEP also reported receiving informational, financial, and practical support from community. Individuals with no moderate or severe symptoms of anxiety provided help to others because of the pandemic. Research has shown that the majority of informal caregivers during the pandemic had experienced burden with more than one in four also experiencing symptoms of depression (Rajovic et al., 2021). Future research should also focus on the long-term mental health of individuals who gave support to their community and assess changes in patterns of anxiety or depression.

In the current study, strained relationships with friends, family, and partners during the first waves of pandemic were also associated with worse temporal patterns of anxiety and depression. Similar findings have been reported for symptoms of depression (James et al., 2022). The presence of quality relationships has been shown to protect against symptoms of depression during the pandemic (Pieh et al., 2020). An association between satisfaction with marital relationship and support for different family policies has also been shown (James et al., 2022). Family policies aimed at reducing the effect of the pandemic on family well-being include paid family leave, flexible payment plans for bills, unemployment benefits, accessibility to counseling and elder care, and housing assistance (Ben Brik, 2020). Government policy-makers in Canada lent financial and economic support to counteract the impact of COVID-19 pandemic on families with various recovery benefits (Chartered Professional Accountants (CPA) Canada, 2021). Future federal policies could also aim at increasing in all provinces accessibility to counseling (marital, parenting, mental health support), external resources for generating potential meaningful relationships in the community, and elder care to mitigate the negative impact of the pandemic on the mental health of Canadians and growing inequities in access to mental health care (Vasiliadis et al., 2021).

The current study has several strengths. It included a large sample of adults and older adults across Canada who contributed information before and during the pandemic, limiting the potential for recall and social desirability bias in the measurement of changes in symptoms of anxiety and depression. Study limitations also need to be considered. In addition to the sampling biases mentioned above, participants who completed the COVID-19 survey were more likely to report MSS-ANXDEP and to be females, older adults, and individuals reporting higher income, compared to those who only completed the 2018 survey. The potential effect on study results is difficult to predict as, on one hand, females are more likely than males to report symptoms of anxiety and depression, while on the other, older adults are less likely to report symptoms than adults, as are those with higher socio-economic status (Mosier et al., 2010; Yeretzian et al., 2023). As we looked at individual changes from prior to the pandemic, we expect the bias to be non-differential. Several independent variables that focused on capturing changes from before to during the pandemic were assessed during the 2020 pandemic survey and therefore may be subject to recall bias. Further, the measure of these variables overlapped with the follow-up measure of MSS-ANXDEP during the pandemic. Interpretation of findings and conclusions are therefore limited to the presence of associations. The study period included the first two waves of the pandemic, and therefore, findings on the temporal patterns may be generalizable to this period of the pandemic. The symptoms of anxiety and depression during the later waves of the pandemic need further study to assess the long-term association and effects of socio-economic and health behaviours on temporal patterns of anxiety and depression. Factors associated with balancing work and childcare obligations due to school closures were not ascertained in this study. Finally, the sample did not include individuals in institutions who may present with more complex mental health problems, and therefore, results may be generalizable to community-living adults and older adults insured under public health systems.

## Conclusion

The present study showed the presence of health and socio-economic inequities associated with persistent, incident, and remitted MSS-ANXDEP from before to during the COVID-19 pandemic. This highlights the need for renewed, coordinated, and interdisciplinary efforts around public health campaigns targeting the promotion of healthy behaviours, as well as the development and implementation of health policies that aim to reduce barriers and improve access to integrated substance use and mental health care within primary and community-based settings.

## Contributions to knowledge

What does this study add to existing literature?

- The current study highlights the presence of sociodemographic and economic factors associated with persistent and incident cases of moderate or severe symptoms of either anxiety or depression, which may increase gaps further with respect to mental health needs.
- The worsening of the quality of relationships with friends, family, and partners during the pandemic was associated with changes in moderate or severe symptoms of either anxiety or depression.
- Health behaviours such as smoking, cannabis use, and drinking more alcohol were associated with changes in moderate or severe symptoms of either anxiety or depression.

What are the key implications for public health interventions, practice, or policy?

- Priorities for population health and value-based health systems include equitable distribution of health. Increased focus should therefore be on those reporting lower income and reduced income from prior to the pandemic, females and gender diverse populations, and essential workers.
- Public health campaigns should reiterate the negative health effects of smoking, cannabis use, and alcohol consumption and provide access to integrated substance and mental health programs.
- Public health policies should be tailored to meet the mental health and service needs of populations that may be more at risk in the aftermath of the pandemic such as those with loss of income, reporting worsening of relationships since the pandemic, and females and gender diverse populations. Reducing economic and social barriers to mental health should be considered.

### Supplementary information

ESM 1 (DOCX 112 kb)

## Data Availability

The authors are not legally authorized to share or publicly publish CanPath data. Participants were not requested to give informed consent for data sharing. Requests for access to the data should be addressed to CanPath.
