# Correction: Factors associated with change in moderate or severe symptoms of anxiety and depression in community-living adults and older adults during the COVID-19 pandemic

**DOI:** 10.17269/s41997-024-00864-y

**Published:** 2024-02-12

**Authors:** Helen-Maria Vasiliadis, Jessica Spagnolo, Mary Bartram, Marie-Josée Fleury, Jean-Philippe Gouin, Sébastien Grenier, Pasquale Roberge, Grace Shen-Tu, Jennifer E. Vena, Catherine Lamoureux-Lamarche, JianLi Wang

**Affiliations:** 1 Département des sciences de la santé communautaire, Université de Sherbrooke, Sherbrooke, Québec Canada; 2 Centre de recherche Charles-Le Moyne, Longueuil, Québec Canada; 3 Mental Health Commission of Canada, Ottawa, Ontario Canada; 4 School of Public Policy & Administration, Carleton University, Ottawa, Ontario Canada; 5 Douglas Mental Health University Institute, Verdun, Québec Canada; 6 McGill University, Montreal, Québec Canada; 7 Department of Psychology, Concordia University, Montreal, Québec Canada; 8 Centre de recherche de l’Institut universitaire de gériatrie de Montréal, Montreal, Québec Canada; 9 Département de psychologie, Université de Montréal, Montréal, Québec Canada; 10 Département de médecine familiale et d’urgence, Université de Sherbrooke, Sherbrooke, Québec Canada; 11 Centre de recherche du Centre hospitalier universitaire de Sherbrooke, Sherbrooke, Québec Canada; 12 Alberta’s Tomorrow Project, Cancer Research & Analytics, Cancer Care Alberta, Alberta Health Services, Calgary, Alberta Canada; 13 Department of Community Health and Epidemiology, Dalhousie University, Halifax, Nova Scotia Canada

**Correction: Canadian Journal of Public Health**

10.17269/s41997-023-00832-y

This article was updated to correct Table 1. The last line of the table (≥ 3 chronic physical conditions) was omitted from the original publication.

