# Supplementary material for: Factors associated with change in moderate or severe symptoms of anxiety and depression in community-living adults and older adults during the COVID-19 pandemic
Source: Can J Public Health. 2023 Dec 20;115(2):230–43. doi: 10.17269/s41997-023-00832-y (PMC11006639; doi:10.17269/s41997-023-00832-y)
Supplement: Supplementary file 1 — (DOCX 112 kb) [file 41997_2023_832_MOESM1_ESM.docx]

**Supplementary Table 1. Multivariable analyses of temporal patterns of MSS of anxiety and depression in adults aged < 65 years**

|  | **Depression or anxiety**  **Moderate or severe symptoms** | | | | | | | | |
| --- | --- | --- | --- | --- | --- | --- | --- | --- | --- |
|  | *Sociodemographic and economic factors* | | | | | | | | |
|  | **Remitted versus no disorder** | | | **Incident versus no disorder** | | | **Persistent versus no disorder** | | |
|  | **AOR** | **95% CI** | | **AOR** | **95% CI** | | **AOR** | **95% CI** | |
| Gender identity |  | | |  | | |  | | |
| Male | **Reference** | | | **Reference** | | | **Reference** | | |
| Female | **1.255** | **1.122** | **1.404** | **1.934** | **1.718** | **2.176** | **1.583** | **1.366** | **1.835** |
| Gender diverse groups | 1.744 | 0.808 | 3.763 | **4.004** | **2.103** | **7.621** | **3.577** | **1.683** | **7.600** |
| Self-reporting as White (yes vs no) | 0.782 | 0.660 | 0.927 | 1.048 | 0.872 | 1.259 | 0.875 | 0.694 | 1.102 |
| Income prior to pandemic |  |  |  |  |  |  |  |  |  |
| Less than $24,999 | **2.127** | **1.649** | **2.742** | 1.015 | 0.763 | 1.351 | **2.480** | **1.859** | **3.308** |
| $25,000–$49,999 | **1.608** | **1.324** | **1.954** | 1.147 | 0.948 | 1.388 | **1.780** | **1.406** | **2.254** |
| $50,000–$74,999 | **1.328** | **1.120** | **1.575** | 1.067 | 0.908 | 1.254 | **1.420** | **1.147** | **1.757** |
| $75,000–$99,999 | **1.201** | **1.018** | **1.416** | 1.080 | 0.928 | 1.256 | **1.489** | **1.216** | **1.823** |
| $100,000–$149,999 | **1.165** | **1.007** | **1.348** | 1.078 | 0.945 | 1.230 | **1.383** | **1.151** | **1.662** |
| $150,000 or more | **Reference** | | | **Reference** | | | **Reference** | | |
| Prefer not to respond | **1.327** | **1.121** | **1.571** | 0.970 | 0.820 | 1.147 | **1.314** | **1.048** | **1.646** |
| Missing response | **1.805** | **1.142** | **2.853** | 1.404 | 0.904 | 2.181 | 1.117 | 0.554 | 2.252 |
| Decrease in income during pandemic | **1.153** | **1.033** | **1.287** | **1.244** | **1.122** | **1.379** | **1.251** | **1.098** | **1.426** |
| Loss of job during the pandemic | 0.970 | 0.809 | 1.163 | 0.893 | 0.756 | 1.053 | 0.865 | 0.697 | 1.072 |
| Work status |  |  |  |  |  |  |  |  |  |
| Full-time or part-time employed/self-employed | **Reference** | | | **Reference** | | | **Reference** | | |
| Retired | **0.756** | **0.654** | **0.874** | **0.660** | **0.567** | **0.767** | **0.540** | **0.441** | **0.661** |
| Looking after home and/or family/ Doing unpaid or voluntary work/student | 1.086 | 0.835 | 1.413 | 0.842 | 0.641 | 1.106 | 1.105 | 0.813 | 1.501 |
| Unable to work because of sickness/disability | **1.854** | **1.470** | **2.337** | **1.817** | **1.440** | **2.291** | **3.293** | **2.646** | **4.097** |
| Unemployed | **1.450** | **1.052** | **1.999** | 1.343 | 0.965 | 1.867 | 1.158 | 0.768 | 1.745 |
| Prefer not to answer/missing data | 1.643 | 0.810 | 3.335 | 1.457 | 0.683 | 3.109 | 1.121 | 0.412 | 3.055 |
|  | *Lifestyle and heatlh behaviours factors* | | | | | | | | |
| Current smoking | **1.339** | **1.117** | **1.607** | **1.218** | **1.017** | **1.457** | **1.511** | **1.240** | **1.840** |
| Past-year cannabis use | **1.141** | **1.009** | **1.289** | **1.571** | **1.410** | **1.751** | **1.720** | **1.509** | **1.962** |
| Past-year average weekly alcohol consumption |  |  |  |  |  |  |  |  |  |
| Never | **Reference** | | | **Reference** | | | **Reference** | | |
| Less than Daily (≤5 times/week) | **0.862** | **0.747** | **0.995** | **0.779** | **0.673** | **0.902** | **0.669** | **0.566** | **0.790** |
| Almost daily (Six to seven times a week) | 0.869 | 0.705 | 1.070 | 0.887 | 0.727 | 1.081 | **0.715** | **0.558** | **0.916** |
| Drinking alcohol more often than March 2020 | 1.111 | 0.986 | 1.252 | **1.688** | **1.523** | **1.871** | **1.627** | **1.419** | **1.865** |
| Medical professional | 0.902 | 0.765 | 1.063 | 1.025 | 0.886 | 1.184 | 1.028 | 0.848 | 1.246 |
| Essential worker | 1.148 | 0.982 | 1.343 | **1.351** | **1.168** | **1.563** | **1.244** | **1.032** | **1.501** |
|  | *Psychosocial factors* | | | | | | | | |
| Provide help to others because of the pandemic | **0.844** | **0.764** | **0.933** | 1.010 | 0.914 | 1.117 | 0.888 | 0.784 | 1.006 |
| Receipt of informational/financial/practical support | 0.902 | 0.804 | 1.013 | **1.395** | **1.261** | **1.543** | 1.074 | 0.943 | 1.224 |
| Change in relationship with friends |  |  |  |  |  |  |  |  |  |
| More distant or strained than before the pandemic | **Reference** | | | **Reference** | | | **Reference** | | |
| About the same as before the pandemic | **0.809** | **0.723** | **0.906** | **0.521** | **0.471** | **0.576** | **0.444** | **0.391** | **0.505** |
| Has become closer than before the pandemic | 0.883 | 0.731 | 1.067 | **0.680** | **0.577** | **0.802** | **0.590** | **0.475** | **0.733** |
| Not applicable/not reported | 1.070 | 0.784 | 1.460 | **0.630** | **0.449** | **0.884** | 0.730 | 0.512 | 1.041 |
| Change in relationship with family |  |  |  |  |  |  |  |  |  |
| More distant or strained than before the pandemic | **Reference** | | | **Reference** | | | **Reference** | | |
| about the same as before the pandemic | **0.810** | **0.694** | **0.947** | **0.498** | **0.439** | **0.565** | **0.478** | **0.410** | **0.558** |
| Has become closer than before the pandemic | **0.689** | **0.574** | **0.828** | **0.539** | **0.464** | **0.625** | **0.435** | **0.360** | **0.526** |
| Not applicable/not reported | 0.832 | 0.624 | 1.108 | **0.545** | **0.410** | **0.723** | **0.323** | **0.226** | **0.461** |
| Change in relationship with partner |  |  |  |  |  |  |  |  |  |
| More distant or strained than before the pandemic | **Reference** | | | **Reference** | | | **Reference** | | |
| About the same as before the pandemic | **0.671** | **0.566** | **0.797** | **0.289** | **0.254** | **0.330** | **0.278** | **0.235** | **0.329** |
| Has become closer than before the pandemic | **0.756** | **0.622** | **0.919** | **0.372** | **0.319** | **0.434** | **0.333** | **0.272** | **0.407** |
| Not applicable/not reported | **0.807** | **0.667** | **0.977** | **0.402** | **0.345** | **0.470** | **0.481** | **0.399** | **0.580** |
|  | *Clinical factors* | | | | | | | | |
| Lifetime physician diagnosis of a mental disorder |  |  |  |  |  |  |  |  |  |
| No | **Reference** | | | **Reference** | | | **Reference** | | |
| Yes | **3.764** | **3.400** | **4.167** | **3.541** | **3.212** | **3.903** | **9.864** | **8.577** | **11.344** |
| Not answered | **4.147** | **1.776** | **9.684** | **3.740** | **1.554** | **9.003** | **8.673** | **3.165** | **23.768** |
| The presence of multimorbidity |  |  |  |  |  |  |  |  |  |
| No chronic physical conditions | **Reference** | | | **Reference** | | | **Reference** | | |
| 1–2 chronic physical conditions | **1.239** | **1.115** | **1.377** | **1.216** | **1.100** | **1.343** | **1.642** | **1.424** | **1.894** |
| ≥3 chronic physical conditions | **2.018** | **1.649** | **2.469** | **2.051** | **1.685** | **2.495** | **3.520** | **2.822** | **4.391** |
| *AOR: adjusted odds ratios; models adjusted for study factors and regional cohorts. | | | | | | | | | |

**Supplementary Table 2. Multivariable analyses of temporal patterns of MSS of anxiety and depression in older adults aged ≥ 65 years**

|  | **Depression or anxiety**  **Moderate or severe symptoms** | | | | | | | | |
| --- | --- | --- | --- | --- | --- | --- | --- | --- | --- |
|  | *Sociodemographic and economic factors* | | | | | | | | |
|  | **Remitted versus no disorder** | | | **Incident versus no disorder** | | | **Persistent versus no disorder** | | |
|  | **AOR** | **95% CI** | | **AOR** | **95% CI** | | **AOR** | **95% CI** | |
| Gender identity Male | **Reference** | | | **Reference** | | | **Reference** | | |
| Female | **1.299** | **1.131** | **1.492** | **2.132** | **1.819** | **2.497** | **1.743** | **1.393** | **2.181** |
| Gender diverse groups | **2.423** | **1.075** | **5.464** | 0.965 | 0.223 | 4.168 | 0.801 | 0.101 | 6.352 |
| Self-reporting as White (yes vs no) | 0.796 | 0.607 | 1.044 | 0.815 | 0.606 | 1.097 | 0.861 | 0.558 | 1.326 |
| Income prior to pandemic |  |  |  |  |  |  |  |  |  |
| Less than $24,999 | **2.689** | **1.886** | **3.833** | **1.467** | **1.006** | **2.140** | **2.215** | **1.323** | **3.707** |
| $25,000–$49,999 | **1.886** | **1.405** | **2.533** | 1.272 | 0.935 | 1.730 | **1.660** | **1.057** | **2.608** |
| $50,000–$74,999 | **1.385** | **1.037** | **1.848** | 1.151 | 0.855 | 1.550 | 1.068 | 0.679 | 1.677 |
| $75,000–$99,999 | 1.219 | 0.906 | 1.641 | 1.177 | 0.870 | 1.592 | 0.959 | 0.598 | 1.537 |
| $100,000–$149,999 | 1.056 | 0.776 | 1.436 | 1.114 | 0.817 | 1.520 | 0.830 | 0.504 | 1.365 |
| $150,000 or more | **Reference** | | | **Reference** | | | **Reference** | | |
| Prefer not to respond | **1.427** | **1.064** | **1.914** | 1.152 | 0.849 | 1.562 | 1.254 | 0.793 | 1.984 |
| Missing response | **2.027** | **1.172** | **3.506** | 1.485 | 0.820 | 2.688 | 2.136 | 0.977 | 4.671 |
| Decrease in income during pandemic | **1.209** | **1.016** | **1.440** | **1.294** | **1.084** | **1.545** | **1.402** | **1.088** | **1.807** |
| Loss of job during the pandemic | 0.920 | 0.615 | 1.377 | 0.780 | 0.504 | 1.207 | 0.789 | 0.423 | 1.473 |
| Work status |  |  |  |  |  |  |  |  |  |
| Full-time or part-time employed/self-employed | **Reference** | | | **Reference** | | | **Reference** | | |
| Retired | 0.852 | 0.699 | 1.040 | 1.035 | 0.836 | 1.280 | 1.042 | 0.760 | 1.428 |
| Looking after home and/or family/ Doing unpaid or voluntary work/student | 0.942 | 0.626 | 1.416 | 1.283 | 0.876 | 1.877 | 0.863 | 0.454 | 1.641 |
| Unable to work because of sickness/disability | 1.735 | 0.718 | 4.191 | 1.874 | 0.754 | 4.658 | **4.104** | **1.765** | **9.540** |
| Unemployed | **2.822** | **1.215** | **6.555** | 0.468 | 0.062 | 3.540 | 1.725 | 0.357 | 8.338 |
| Prefer not to answer/missing data | 1.826 | 0.516 | 6.459 | 0.615 | 0.078 | 4.877 | 2.412 | 0.489 | 11.894 |
|  | *Lifestyle and heatlh behaviour factors* | | | | | | | | |
| Current smoking | 1.064 | 0.767 | 1.476 | 1.343 | 0.980 | 1.839 | **2.334** | **1.644** | **3.314** |
| Past-year cannabis use | **1.431** | **1.196** | **1.713** | **1.659** | **1.390** | **1.981** | **1.461** | **1.134** | **1.882** |
| Past-year average weekly alcohol consumption |  |  |  |  |  |  |  |  |  |
| Never | **Reference** | | | **Reference** | | | **Reference** | | |
| Less than Daily (≤5 times/week) | 0.894 | 0.747 | 1.070 | **0.746** | **0.619** | **0.898** | **0.675** | **0.527** | **0.863** |
| Almost daily (Six to seven times a week) | 0.849 | 0.676 | 1.066 | **0.768** | **0.607** | **0.971** | 0.764 | 0.551 | 1.059 |
| Drinking alcohol more often than March 2020 | 1.166 | 0.970 | 1.402 | **1.496** | **1.260** | **1.776** | 1.154 | 0.880 | 1.514 |
| Medical professional | 1.083 | 0.659 | 1.779 | **0.426** | **0.203** | **0.894** | 1.041 | 0.477 | 2.271 |
| Essential worker | 1.383 | 0.946 | 2.024 | **1.533** | **1.007** | **2.333** | 1.082 | 0.548 | 2.135 |
|  | *Psychosocial factors* | | | | | | | | |
| Provide help to others because of the pandemic | **0.808** | **0.711** | **0.919** | 0.939 | 0.817 | 1.080 | **0.744** | **0.612** | **0.904** |
| Receipt of informational/financial/practical support | **0.747** | **0.646** | **0.863** | **1.260** | **1.100** | **1.444** | 1.002 | 0.819 | 1.226 |
| Change in relationship with friends |  |  |  |  |  |  |  |  |  |
| More distant or strained than before the pandemic | **Reference** | | | **Reference** | | | **Reference** | | |
| about the same as before the pandemic | 0.957 | 0.806 | 1.136 | **0.428** | **0.366** | **0.500** | **0.406** | **0.325** | **0.508** |
| Has become closer than before the pandemic | 0.950 | 0.737 | 1.225 | **0.513** | **0.408** | **0.645** | **0.574** | **0.409** | **0.805** |
| Not applicable/not reported | **1.488** | **1.050** | **2.109** | 0.777 | 0.536 | 1.126 | 0.975 | 0.621 | 1.532 |
| Change in relationship with family |  |  |  |  |  |  |  |  |  |
| More distant or strained than before the pandemic | **Reference** | | | **Reference** | | | **Reference** | | |
| about the same as before the pandemic | **0.680** | **0.549** | **0.843** | **0.552** | **0.455** | **0.669** | **0.526** | **0.405** | **0.683** |
| Has become closer than before the pandemic | 0.819 | 0.635 | 1.055 | **0.787** | **0.627** | **0.986** | **0.482** | **0.342** | **0.677** |
| Not applicable/not reported | 0.892 | 0.666 | 1.196 | **0.638** | **0.475** | **0.856** | **0.614** | **0.418** | **0.902** |
| Change in relationship with partner |  |  |  |  |  |  |  |  |  |
| More distant or strained than before the pandemic | **Reference** | | | **Reference** | | | **Reference** | | |
| about the same as before the pandemic | **0.540** | **0.422** | **0.693** | **0.224** | **0.182** | **0.277** | **0.202** | **0.151** | **0.270** |
| Has become closer than before the pandemic | **0.514** | **0.386** | **0.684** | **0.278** | **0.218** | **0.356** | **0.226** | **0.158** | **0.325** |
| Not applicable/not reported | **0.518** | **0.398** | **0.675** | **0.344** | **0.276** | **0.429** | **0.297** | **0.219** | **0.402** |
|  | *Clinical factors* | | | | | | | | |
| Lifetime physician diagnosis of a mental disorder |  |  |  |  |  |  |  |  |  |
| No | **Reference** | | | **Reference** | | | **Reference** | | |
| Yes | **4.265** | **3.737** | **4.867** | **4.582** | **3.999** | **5.249** | **10.363** | **8.390** | **12.800** |
| Not answered | **13.127** | **6.090** | **28.296** | 1.058 | 0.138 | 8.118 | 3.391 | 0.433 | 26.580 |
| The presence of multimorbidity |  |  |  |  |  |  |  |  |  |
| No chronic physical conditions | **Reference** | | | **Reference** | | | **Reference** | | |
| 1–2 chronic physical conditions | **1.474** | **1.220** | **1.782** | **1.661** | **1.346** | **2.049** | **1.559** | **1.119** | **2.172** |
| ≥3 chronic physical conditions | **2.522** | **1.985** | **3.204** | **2.321** | **1.785** | **3.018** | **3.617** | **2.496** | **5.243** |
| *AOR: adjusted odds ratios; models adjusted for study factors and regional cohorts. | | | | | | | | | |

**Supplementary Table 3. Multivariable analyses of temporal patterns of MSS of anxiety and depression in females**

|  | **Depression or anxiety**  **Moderate or severe symptoms** | | | | | | | | |
| --- | --- | --- | --- | --- | --- | --- | --- | --- | --- |
|  | *Sociodemographic and economic factors* | | | | | | | | |
|  | **Remitted versus no disorder** | | | **Incident versus no disorder** | | | **Persistent versus no disorder** | | |
|  | **AOR** | **95% CI** | | **AOR** | **95% CI** | | **AOR** | **95% CI** | |
| Age groups 35–44 years | **2.268** | **1.825** | **2.820** | **2.305** | **1.897** | **2.802** | **4.363** | **3.396** | **5.605** |
| 45–54 years | **1.844** | **1.574** | **2.160** | **1.765** | **1.522** | **2.047** | **2.986** | **2.445** | **3.646** |
| 55–64 years | **1.396** | **1.235** | **1.577** | **1.362** | **1.211** | **1.532** | **1.586** | **1.342** | **1.874** |
| ≥65 years | **Reference** | | | **Reference** | | | **Reference** | | |
| Self-reporting as White (yes vs no) | **0.785** | **0.657** | **0.939** | 1.013 | 0.842 | 1.220 | 0.910 | 0.714 | 1.160 |
| Income prior to pandemic |  |  |  |  |  |  |  |  |  |
| Less than $24,999 | **2.348** | **1.860** | **2.965** | 1.212 | 0.956 | 1.538 | **2.840** | **2.154** | **3.745** |
| $25,000–$49,999 | **1.628** | **1.357** | **1.954** | 1.140 | 0.963 | 1.350 | **1.839** | **1.461** | **2.315** |
| $50,000–$74,999 | **1.307** | **1.102** | **1.549** | 1.050 | 0.901 | 1.224 | **1.470** | **1.185** | **1.824** |
| $75,000–$99,999 | **1.200** | **1.012** | **1.423** | 1.021 | 0.878 | 1.186 | **1.445** | **1.168** | **1.788** |
| $100,000–$149,999 | 1.150 | 0.981 | 1.349 | 1.057 | 0.921 | 1.213 | **1.323** | **1.082** | **1.617** |
| $150,000 or more | **Reference** | | | **Reference** | | | **Reference** | | |
| Prefer not to respond | **1.439** | **1.220** | **1.697** | 0.988 | 0.845 | 1.155 | **1.496** | **1.199** | **1.867** |
| Missing response | **2.010** | **1.392** | **2.903** | 1.354 | 0.936 | 1.960 | **1.762** | **1.043** | **2.976** |
| Decrease in income during pandemic | **1.250** | **1.122** | **1.393** | **1.277** | **1.156** | **1.411** | **1.263** | **1.108** | **1.440** |
| Loss of job during the pandemic | 0.847 | 0.694 | 1.035 | 0.856 | 0.719 | 1.020 | **0.742** | **0.585** | **0.943** |
| Work status |  |  |  |  |  |  |  |  |  |
| Full-time or part-time employed/self-employed | **Reference** | | | **Reference** | | | **Reference** | | |
| Retired | 0.881 | 0.770 | 1.008 | 0.933 | 0.820 | 1.060 | 0.861 | 0.720 | 1.030 |
| Looking after home and/or family/ Doing unpaid or voluntary work/student | 1.046 | 0.825 | 1.325 | 0.913 | 0.721 | 1.155 | 1.081 | 0.813 | 1.439 |
| Unable to work because of sickness/disability | **2.010** | **1.573** | **2.569** | **1.795** | **1.404** | **2.294** | **3.145** | **2.482** | **3.987** |
| Unemployed | **1.547** | **1.059** | **2.260** | 1.293 | 0.875 | 1.911 | 1.131 | 0.696 | 1.837 |
| Prefer not to answer/missing data | 1.313 | 0.632 | 2.729 | 0.961 | 0.419 | 2.208 | 1.235 | 0.489 | 3.118 |
|  | *Lifestyle and heatlh behaviour factors* | | | | | | | | |
| Current smoking | **1.359** | **1.127** | **1.639** | **1.298** | **1.087** | **1.550** | **1.787** | **1.468** | **2.175** |
| Past-year cannabis use | **1.161** | **1.026** | **1.314** | **1.531** | **1.376** | **1.703** | **1.544** | **1.350** | **1.766** |
| Past-year average weekly alcohol consumption |  |  |  |  |  |  |  |  |  |
| Never | **Reference** | | | **Reference** | | | **Reference** | | |
| Less than Daily (≤5 times/week) | **0.873** | **0.765** | **0.996** | **0.782** | **0.687** | **0.890** | **0.703** | **0.600** | **0.824** |
| Almost daily (Six to seven times a week) | 0.905 | 0.752 | 1.090 | 0.869 | 0.731 | 1.035 | 0.807 | 0.642 | 1.015 |
| Drinking alcohol more often than March 2020 | 1.039 | 0.923 | 1.169 | **1.578** | **1.430** | **1.742** | **1.460** | **1.275** | **1.672** |
| Medical professional | 0.920 | 0.776 | 1.090 | 0.999 | 0.859 | 1.163 | 0.945 | 0.769 | 1.160 |
| Essential worker | 1.128 | 0.943 | 1.349 | **1.307** | **1.112** | **1.537** | 1.207 | 0.978 | 1.490 |
|  | *Psychosocial factors* | | | | | | | | |
| Provide help to others because of the pandemic | **0.852** | **0.775** | **0.936** | 1.023 | 0.932 | 1.123 | 0.891 | 0.790 | 1.005 |
| Receipt of informational/financial/practical support | **0.820** | **0.739** | **0.911** | **1.314** | **1.201** | **1.438** | 0.962 | 0.850 | 1.088 |
| Change in relationship with friends |  |  |  |  |  |  |  |  |  |
| More distant or strained than before the pandemic | **Reference** | | | **Reference** | | | **Reference** | | |
| about the same as before the pandemic | **0.830** | **0.743** | **0.927** | **0.490** | **0.446** | **0.539** | **0.419** | **0.370** | **0.474** |
| Has become closer than before the pandemic | 0.897 | 0.757 | 1.063 | **0.610** | **0.527** | **0.705** | **0.552** | **0.453** | **0.673** |
| Not applicable/not reported | 1.301 | 0.976 | 1.735 | 0.780 | 0.583 | 1.043 | 0.878 | 0.632 | 1.219 |
| Change in relationship with family |  |  |  |  |  |  |  |  |  |
| More distant or strained than before the pandemic | **Reference** | | | **Reference** | | | **Reference** | | |
| about the same as before the pandemic | **0.693** | **0.599** | **0.803** | **0.482** | **0.428** | **0.543** | **0.471** | **0.405** | **0.548** |
| Has become closer than before the pandemic | **0.678** | **0.572** | **0.803** | **0.565** | **0.492** | **0.649** | **0.448** | **0.373** | **0.539** |
| Not applicable/not reported | **0.745** | **0.584** | **0.952** | **0.530** | **0.421** | **0.667** | **0.417** | **0.310** | **0.561** |
| Change in relationship with partner |  |  |  |  |  |  |  |  |  |
| More distant or strained than before the pandemic | **Reference** | | | **Reference** | | | **Reference** | | |
| about the same as before the pandemic | **0.652** | **0.550** | **0.772** | **0.279** | **0.245** | **0.317** | **0.269** | **0.227** | **0.319** |
| Has become closer than before the pandemic | **0.643** | **0.529** | **0.781** | **0.368** | **0.317** | **0.427** | **0.309** | **0.252** | **0.379** |
| Not applicable/not reported | **0.674** | **0.562** | **0.809** | **0.394** | **0.342** | **0.455** | **0.443** | **0.370** | **0.531** |
|  | *Clinical factors* | | | | | | | | |
| Lifetime physician diagnosis of a mental disorder |  |  |  |  |  |  |  |  |  |
| No | **Reference** | | | **Reference** | | | **Reference** | | |
| Yes | **3.647** | **3.319** | **4.008** | **3.639** | **3.331** | **3.977** | **9.519** | **8.328** | **10.881** |
| Not answered | **5.257** | **2.561** | **10.791** | 1.985 | 0.749 | 5.258 | **8.217** | **3.261** | **20.702** |
| The presence of multimorbidity |  |  |  |  |  |  |  |  |  |
| No chronic physical conditions | **Reference** | | | **Reference** | | | **Reference** | | |
| 1–2 chronic physical conditions | **1.289** | **1.158** | **1.435** | **1.330** | **1.203** | **1.471** | **1.872** | **1.611** | **2.176** |
| ≥3 chronic physical conditions | **2.175** | **1.830** | **2.585** | **2.114** | **1.790** | **2.497** | **4.204** | **3.415** | **5.176** |
| *AOR: adjusted odds ratios; models adjusted for study factors and regional cohorts. | | | | | | | | | |

**Supplementary Table 4. Multivariable analyses of temporal patterns of MSS of anxiety and depression in males**

|  | **Depression or anxiety**  **Moderate or severe symptoms** | | | | | | | | |
| --- | --- | --- | --- | --- | --- | --- | --- | --- | --- |
|  | *Sociodemographic and economic factors* | | | | | | | | |
|  | **Remitted versus no disorder** | | | **Incident versus no disorder** | | | **Persistent versus no disorder** | | |
|  | **AOR** | **95% CI** | | **AOR** | **95% CI** | | **AOR** | **95% CI** | |
| Age groups 35–44 years | **1.775** | **1.173** | **2.685** | **2.500** | **1.677** | **3.725** | **3.694** | **2.174** | **6.278** |
| 45–54 years | **2.327** | **1.822** | **2.972** | **2.241** | **1.695** | **2.963** | **3.890** | **2.700** | **5.605** |
| 55–64 years | **1.420** | **1.173** | **1.719** | **1.403** | **1.117** | **1.763** | **1.680** | **1.233** | **2.288** |
| ≥65 years | **Reference** | | | **Reference** | | | **Reference** | | |
| Self-reporting as White (yes vs no) | 0.828 | 0.645 | 1.063 | 0.957 | 0.710 | 1.290 | 0.888 | 0.601 | 1.313 |
| Income prior to pandemic |  |  |  |  |  |  |  |  |  |
| Less than $24,999 | **2.905** | **1.972** | **4.280** | 1.198 | 0.719 | 1.994 | **2.667** | **1.570** | **4.531** |
| $25,000–$49,999 | **2.559** | **1.930** | **3.394** | 1.397 | 0.985 | 1.981 | **2.975** | **1.972** | **4.488** |
| $50,000–$74,999 | **1.742** | **1.350** | **2.249** | 1.238 | 0.922 | 1.663 | 1.369 | 0.916 | 2.047 |
| $75,000–$99,999 | **1.393** | **1.081** | **1.794** | **1.423** | **1.084** | **1.868** | 1.397 | 0.951 | 2.053 |
| $100,000–$149,999 | 1.194 | 0.944 | 1.511 | 1.156 | 0.899 | 1.486 | 1.370 | 0.969 | 1.936 |
| $150,000 or more | **Reference** | | | **Reference** | | | **Reference** | | |
| Prefer not to respond | 1.302 | 0.968 | 1.750 | 1.290 | 0.927 | 1.795 | **1.607** | **1.026** | **2.517** |
| Missing response | 1.527 | 0.540 | 4.321 | 1.993 | 0.693 | 5.731 | 1.100 | 0.144 | 8.380 |
| Decrease in income during pandemic | 1.061 | 0.888 | 1.269 | **1.287** | **1.059** | **1.565** | **1.386** | **1.076** | **1.784** |
| Loss of job during the pandemic | 1.253 | 0.932 | 1.684 | 0.923 | 0.665 | 1.281 | 1.351 | 0.911 | 2.003 |
| Work status |  |  |  |  |  |  |  |  |  |
| Full-time or part-time employed/self-employed | **Reference** | | | **Reference** | | | **Reference** | | |
| Retired | **0.779** | **0.630** | **0.963** | 0.788 | 0.613 | 1.014 | 0.939 | 0.668 | 1.319 |
| Looking after home and/or family/ Doing unpaid or voluntary work/student | 0.762 | 0.409 | 1.421 | 1.299 | 0.730 | 2.312 | 0.346 | 0.102 | 1.170 |
| Unable to work because of sickness/disability | 1.382 | 0.809 | 2.362 | **2.335** | **1.361** | **4.008** | **5.970** | **3.685** | **9.670** |
| Unemployed | 1.450 | 0.874 | 2.407 | 1.359 | 0.759 | 2.434 | 1.265 | 0.611 | 2.617 |
| Prefer not to answer/missing data | **3.411** | **1.018** | **11.430** | **4.020** | **1.065** | **15.175** | 1.442 | 0.152 | 13.718 |
|  | *Lifestyle and heatlh behaviour factors* | | | | | | | | |
| Current smoking | 1.015 | 0.749 | 1.374 | 1.132 | 0.812 | 1.579 | 1.309 | 0.895 | 1.915 |
| Past-year cannabis use | **1.307** | **1.089** | **1.567** | **1.691** | **1.394** | **2.052** | **1.714** | **1.343** | **2.187** |
| Past-year average weekly alcohol consumption |  |  |  |  |  |  |  |  |  |
| Never | **Reference** | | | **Reference** | | | **Reference** | | |
| Less than Daily (≤5 times/week) | 0.875 | 0.705 | 1.084 | **0.704** | **0.547** | **0.906** | **0.595** | **0.444** | **0.798** |
| Almost daily (Six to seven times a week) | 0.810 | 0.616 | 1.066 | 0.762 | 0.556 | 1.044 | **0.660** | **0.443** | **0.983** |
| Drinking alcohol more often than March 2020 | **1.329** | **1.099** | **1.608** | **1.740** | **1.428** | **2.121** | **1.452** | **1.102** | **1.913** |
| Medical professional | 0.803 | 0.525 | 1.229 | 0.900 | 0.589 | 1.374 | 1.396 | 0.862 | 2.259 |
| Essential worker | **1.287** | **1.002** | **1.652** | **1.595** | **1.220** | **2.084** | 1.209 | 0.838 | 1.745 |
|  | *Psychosocial factors* | | | | | | | | |
| Provide help to others because of the pandemic | **0.817** | **0.707** | **0.945** | 0.901 | 0.760 | 1.067 | **0.751** | **0.603** | **0.934** |
| Receipt of informational/financial/practical support | 0.877 | 0.733 | 1.049 | **1.366** | **1.133** | **1.647** | **1.348** | **1.060** | **1.714** |
| Change in relationship with friends |  |  |  |  |  |  |  |  |  |
| More distant or strained than before the pandemic | **Reference** | | | **Reference** | | | **Reference** | | |
| about the same as before the pandemic | 0.926 | 0.773 | 1.108 | **0.514** | **0.426** | **0.620** | **0.564** | **0.441** | **0.722** |
| Has become closer than before the pandemic | 0.942 | 0.673 | 1.321 | 0.773 | 0.549 | 1.091 | 1.009 | 0.642 | 1.586 |
| Not applicable/not reported | 1.054 | 0.707 | 1.572 | **0.591** | **0.361** | **0.967** | 0.774 | 0.446 | 1.344 |
| Change in relationship with family |  |  |  |  |  |  |  |  |  |
| More distant or strained than before the pandemic | **Reference** | | | **Reference** | | | **Reference** | | |
| about the same as before the pandemic | 0.998 | 0.777 | 1.282 | **0.607** | **0.482** | **0.764** | **0.511** | **0.386** | **0.677** |
| Has become closer than before the pandemic | 0.885 | 0.651 | 1.204 | **0.715** | **0.534** | **0.958** | **0.361** | **0.241** | **0.541** |
| Not applicable/not reported | 1.285 | 0.892 | 1.851 | 0.736 | 0.488 | 1.109 | **0.485** | **0.290** | **0.811** |
| Change in relationship with partner |  |  |  |  |  |  |  |  |  |
| More distant or strained than before the pandemic | **Reference** | | | **Reference** | | | **Reference** | | |
| about the same as before the pandemic | **0.611** | **0.470** | **0.794** | **0.245** | **0.195** | **0.306** | **0.219** | **0.163** | **0.294** |
| Has become closer than before the pandemic | 0.765 | 0.569 | 1.029 | **0.253** | **0.191** | **0.335** | **0.287** | **0.201** | **0.412** |
| Not applicable/not reported | 0.855 | 0.634 | 1.154 | **0.405** | **0.304** | **0.539** | **0.420** | **0.295** | **0.597** |
|  | *Clinical factors* | | | | | | | | |
| Lifetime physician diagnosis of a mental disorder |  |  |  |  |  |  |  |  |  |
| No | **Reference** | | | **Reference** | | | **Reference** | | |
| Yes | **4.731** | **4.048** | **5.529** | **4.705** | **3.939** | **5.620** | **11.587** | **9.083** | **14.783** |
| Not answered | **12.687** | **4.941** | **32.575** | **4.613** | **1.017** | **20.925** | **-** | **-** | **-** |
| The presence of multimorbidity |  |  |  |  |  |  |  |  |  |
| No chronic physical conditions | **Reference** | | | **Reference** | | | **Reference** | | |
| 1–2 chronic physical conditions | **1.509** | **1.256** | **1.813** | **1.425** | **1.157** | **1.755** | **1.556** | **1.166** | **2.076** |
| ≥3 chronic physical conditions | **2.707** | **2.036** | **3.598** | **2.269** | **1.624** | **3.171** | **4.246** | **2.869** | **6.283** |
| *AOR: adjusted odds ratios; models adjusted for study factors and regional cohorts. | | | | | | | | | |
